# Perfusion development and its potential for cell therapy manufacturing with adherent cells

**DOI:** 10.1007/s00253-026-13939-2

**Published:** 2026-07-03

**Authors:** Samuel Lukas Schneider, Vivian Ott, Regine Eibl

**Affiliations:** School of Life Sciences and Facility Management, Institute of Chemistry and Biotechnology, ZHAW Zurich University of Applied Sciences, 8820 Wädenswil, Switzerland

**Keywords:** Cell retention device, Stem cell, Perfusion, Process intensification, Cell washing, Single-use technologies

## Abstract

**Abstract:**

Perfusion processing has established itself as a powerful intensification strategy for biopharmaceutical production processes of recombinant proteins. However, in the relatively young field of cell therapeutics manufacturing, this tool has been overlooked so far and with rising approvals, this field is in dire need of scalable and efficient production processes. In this regard, perfusion operation mode and related cell retention devices can be used to support upstream and downstream processing by automating operations, reducing contamination risk and ensuring stable cell quality. In this review, the history of cell retention technologies developed for suspension cell processing is laid out alongside the perfusion-based process intensification strategies they made possible. It is summarized how these technologies could be used to intensify the upstream processing of therapeutic adherent cells and to what degree perfusion processing of these demanding cells is already described in literature. Additionally, the applicability of cell retention devices for harvesting and downstream processing of therapeutic cells, which mainly consists of cell washing and formulation steps, is elaborated. In conclusion, through the implementation of scalable, single-use, good manufacturing practices compliant cultivation systems and cell retention devices, it should be possible substantially to accelerate the development of therapeutic adherent cell manufacturing strategies.

**Key points:**

• *Perfusion can automate medium exchanges in therapeutic adherent cell manufacturing*

• * Perfusion technologies from suspension cell processing can be translated to adherent cell manufacturing*

• *Scalable single-use perfusion systems are needed for the production of cell therapies*

## Introduction

Cell therapeutics are a rapidly growing group of medicinal products that promise to treat a multitude of diseases including cancer (e.g., lymphoma and prostate cancer), autoimmune disorders (e.g., systemic lupus erythematosus, Crohn’s disease, type 1 diabetes), cardiovascular diseases, and neurological disorders (El-Kadiry et al. 2021; Hoang et al. 2022; Hwang and Muzammil 2025). Given their broad applicability and novel curative approaches, it is not surprising that the market size is expected to grow from $5.89 billion in 2025 to $20 billion by 2030 (Cell Therapy Market Size, Share And Growth Report, 2030 2024). Another novel therapeutics group which is often mentioned in the same sentence as cell therapies is gene therapies, which have the aim of fixing genetic malignancies through gene editing. While a distinction is made between these two categories, it is not clear how to classify ex vivo genetically modified cells like chimeric antigen receptor (CAR) T cells for cancer treatment or engineered hematopoietic stem cells (HSCs) for treating sickle cell disease (Hwang and Muzammil 2025). Regardless of the classification, these therapeutical approaches face the challenge that large numbers of sensitive and often anchorage dependent cells need to be produced for each treated patient. For example, in the case of mesenchymal stromal cell (MSC)-based therapeutics, 10^6^ – 10^9^ cells are required per patient and dose (Kabat et al. 2020), which corresponds to cultivation volumes of a few milliliters to liters. While benchtop bioreactors should be sufficient for most autologous therapies, substantially larger bioreactors with volumes of 100 s to 1000 s of liters are required for allogeneic approaches (Scibona and Morbidelli 2019). Therefore, to make these advanced therapy medicinal products available to patients in need at a reasonable cost, efficient and scalable upstream processing (USP) strategies are required, which is where bioprocess intensification comes in. Contrary to regular process optimization, where process parameters are gradually improved, intensification is achieved through major process changes like combining unit operations, switching from batch to continuous operation or modifying production organisms (Boodhoo et al. 2022). As the cells themselves are the final product in cell therapeutics, it is not possible to heavily modify them for increased growth or robustness, limiting the available intensification strategies. Additionally, process intensification approaches have not yet been systematically evaluated for the manufacturing of these relatively novel products.

In the field of recombinant protein production with mammalian host cells, perfusion mode operation has established itself as a valuable USP intensification approach. Perfusion distinguishes itself by continuously renewing the cells’ growth medium, ensuring that they are always provided with sufficient nutrients while waste products are removed (Pörtner 2023). A key aspect of perfusion cultivation is that a cell retention device (CRD) is used to keep the cells in the bioreactor, making it possible to reach cell densities in excess of 10^8^ cells mL^−1^ (Wong et al. 2022). Through process intensification, smaller bioreactors can be used to achieve the same process output as traditional approaches, making it possible to use the advantages of single-use technology throughout the whole production process (Malla et al. 2021; Teale et al. 2026). For example, in the 1990 s, standard antibody production processes using Chinese hamster ovary (CHO) cells were conducted at a scale of 10–20 m^3^ while in the last decade smaller bioreactors with a volume of 2 m^3^ were favored (BioPlan Associates 2025; Croughan et al. 2015).

In this mini review, the history of perfusion processing and the related advances in cell retention technologies are briefly summarized before the focus is put on its challenges and potential for scalable therapeutic adherent (stem) cell manufacturing.

## History of perfusion and cell retention technologies

Perfusion mode cultivation of mammalian cells has a long history and was already applied in the 1950 s in chemostats (Wong et al. 2022). In the 1980 s, early adopters of continuous perfusion were primarily biopharmaceutical companies producing unstable proteins as this process mode allows for continuous harvest and purification (Croughan et al. 2015). As the cultivated cells were predominantly adherent and often immobilized in a fixed-bed, no specialized CRDs were required. In the case of suspended carriers, simple CRDs like spin filters and gravity settlers were used (Bielser et al. 2018). This is still the case for MSCs and induced pluripotent stem cells (iPSCs) cultivated on microcarriers or as aggregates as these particles are substantially larger than cells, simplifying the implementation of sedimentation- and filtration-based cell retention approaches. In the field of tissue engineering, the term perfusion is also used to describe the active pumping of growth medium through 3D constructs, akin to blood flowing through organs in the body (Thomas et al. 2000). This type of perfusion is not the focus of this mini review as it is usually disconnected from medium renewal and in engineering terms could be classified as (repeated-) batch cultivation in fixed-bed bioreactors.

**Fig. 1:**
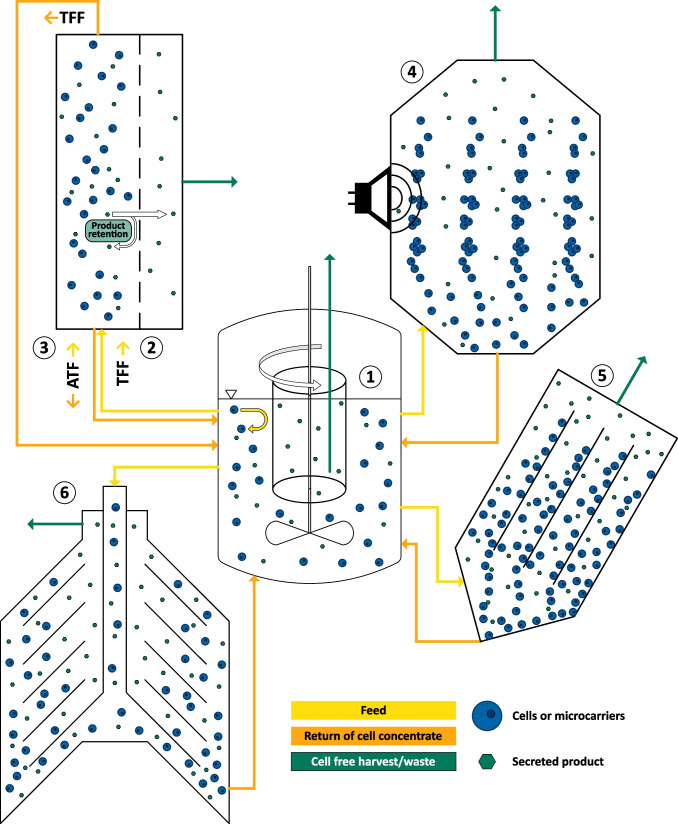
Schematic representation of different CRDs. (**1**) Bioreactor with axially mounted spin filter, (**2**) TFF, (**3**) ATF, (**4**) acoustic settler, (**5**) Inclined settler, and (**6**) continuous disk stack centrifuge

What made it possible to implement perfusion technology to non-immobilized cell processing was the development of a wide variety of CRDs with widely differing operating principles. Broadly, CRDs can be grouped into filtration and settling-based technologies whereby the latter category can be further split into gravitational, centrifugal, and acoustic settling (MacDonald et al. 2022). Figure 1 contains schematic representations of six common CRDs of these groups (Castilho and Medronho 2002).

An ideal CRD features high separation efficiency, exposes the cell to minimal shear, and has low product retention. To facilitate process development, the CRD needs to be scalable, i.e., scale-down models and large version for production-size bioreactors must be available. For continuous production processes, it is crucial that the CRD operates in a simple and robust way, ensuring that processes can run for multiple months without the CRD deteriorating or even developing leaks. As no CRD can fulfill all these demands, the choice of the most suitable CRD is highly dependent on the process needs. Table 1 provides an overview of the strengths and weaknesses of the main CRD types, most of which are installed outside of the bioreactor.

**Table 1:** Operating principle and comparison of different CRDs for perfusion processes. Bioreactors with inherent cell retention (e.g., hollow fiber or fixed-bed bioreactors) are not included

CRD	TFF	ATF	Acoustic settler	Spin filter	Centrifuge	Inclined settler
Sources	Woodside et al. 1998; Castilho and Medronho 2002; Clincke et al. 2013; Chotteau 2015; Wang et al. 2017; Karst et al. 2020; Raza et al. 2024	Chotteau 2015; Wang et al. 2017; Gränicher et al. 2020; Madsen and Brau 2021; Veje et al. 2024; Repligen 2025	Coakley et al. 1994; Trampler et al. 1994; Ryll et al. 2000; Gorenflo et al. 2002, 2003; Shirgaonkar et al. 2004; Chotteau 2015; Venereo Sanchez et al. 2017; Gränicher et al. 2020; Altenburg et al. 2025	Varecka and Scheirer 1987; Avgerinos et al. 1990; Deo et al. 1996; Iding et al. 2000; Castilho and Medronho 2002; Voisard et al. 2003; Bonham-Carter and Shevitz 2011; Pollock et al. 2013; Chotteau 2015; Bosco et al. 2017	Jäger 1992; Johnson et al. 1996; Takamatsu et al. 1996; Woodside et al. 1998; Castilho and Medronho 2002; Hutchinson et al. 2006; Leung 2007; Kim et al. 2008; Chotteau 2015; Baust et al. 2025	Acrivos and Herbolzheimer 1979; Knaack et al. 1994; Searles et al. 1994; Woodside et al. 1998; Castilho and Medronho 2002; Voisard et al. 2003; Vogel et al. 2012; Pohlscheidt et al. 2013; Castilho 2014; Chotteau 2015; Coronel et al. 2020; Glascock and Kompala 2024
Separation principle	Size - filtration	Size - filtration	Density, compressibility and size - acoustic field	Size - filtration	Density and size - centrifugal force	Density and size - gravitational settling
Operating principle	Tangential flow of the cell suspension along a microfiltration membrane; the cell-free medium is removed, and the slightly concentrated cell suspension is returned to the reactor	Tangential flow with alternating direction through a hollow fiber microfiltration module generated by a diaphragm pump	Generation of an ultrasonic standing wave, particles are pushed to pressure nodes of acoustic field where they aggregate and subsequently sediment	Rotating cylindrical filter screen inside bioreactor; separation based on various forces, e.g., centrifugal, radial and steric effects; the clarified medium is removed from the interior of the spin filter	Separation of cells and medium using centrifugal force based on differences in density and size; cells are moved radially outward, cell-free medium is removed from the center of the centrifuge	Cell suspension flows in a laminar fashion between inclined plates; the cells sediment and settle at the bottom of the settler while the cell-free medium is removed at the top of the inclined settler
Disposable or reusable	Disposable and reusable	Disposable and reusable	Disposable and reusable	Reusable	Disposable and reusable	Disposable and reusable
Cell retention	100%	100%	90–99%	75–99%	> 99%	85–99%
Product sieving (− − = strong sieving, + + = weak sieving)	− −	−	+ +	− −	+ +	+ +
Maximum bioreactor volume	Up to 3 000 L reported	Up to 1 000 L reported*	Up to 100 L reported	Up to 500 L (internal) or 1 000 L (external) reported	Up to 2 000 L reported	Up to 3 000 L reported
Perfusion flow rate	Typically up to 2 000 L d^−1^ with flux of ≤ 5 L m^−2^ h^−1^ and 16.6 m^2^ hollow fiber module	Up to 1 500 L d^−1^ with flux of ≤ 5.7 L m^−2^ h^−1^*	Up to 1 000 L d^−1^*	Up to 600 L d^−1^	Up to 2 880 L d^−1^ for Centritech	Up to 2 950 L d^−1^
Internal or external device	External	External	External	Internal and external	External	Internal and external
Maximum reported density of suspension cells [10^6 ^cells mL^−1^]	214	230	60	74	20	60
Residence time in external loop with CRD	< 2 min	< 2 min	< 15 min	No external loop	< 10 min	≥ 30 min
Advantages	- Full cell retention - Scalability	- Full cell retention - Off-the-shelf - Only one bioreactor port necessary - No stress through pumps - Reduced fouling compared to TFF	- No product retention - No fouling - Removal of dead cells and cell debris possible	- Internal CRD, therefore no nutrient limitations (e.g., O₂)	- Can handle high solid contents - No product retention - Short residence time in CRD - Removal of dead cells and cell debris possible	- Low shear stress - No product retention - Removal of dead cells and cell debris possible
Disadvantages	- High product retention - Fouling and potential clogging with increasing viscosity	- High product retention - Fouling and potential clogging with increasing viscosity	- Separation efficiency strongly depends on settings - Challenging to develop	- High space requirement of CRD in the bioreactor - Limited lifetime due to clogging (cells are growing on screen) - Increased fouling at high perfusion rates	- High shear stress possible - Rotating parts and mechanical seals are challenging for long term operation	- Long residence time in CRD (> 1 h) - High blockage risk - High footprint
Supplier	Cytiva, Sartorius, Repligen, Asahi Kasei	Repligen, Cobetter	Ipratech, Getinge	Eppendorf, B. Braun Biotech, Bioengineering	CARR Biosystems, GEA Westfalia Separator Group	Sudhin Biopharma, Biotechnology Solutions
Potential for modern production processes^†^ (+ + = high, − − = low):						
Monoclonal antibody	+ +	+ +	−	− −	+	− −
Virus	− −	− −	+ +	− −	+ +	+ +
Therapeutic adherent cells	−	+	+	− −	− −	−

^*According to manufacturer, †authors’ opinion on the suitability of the CRDs for manufacturing the listed products in perfusion mode^

The spin filter represents an early filtration-based CRD which consists of a rotating filter cage, typically mounted inside the bioreactor on the same axis as the agitator; however, external versions exist. While filter fouling is reduced by the relative fluid motion induced through the spinning filter, the reliance on moving parts that penetrate the sterile barrier increased contamination risk and made them difficult to replace in case of malfunctions (Woodside et al. 1998). Starting in the 2010 s, there was a shift to tangential flow filtration (TFF) technologies, which are now the predominant cell retention technologies (MacDonald et al. 2022). Here, the cell suspension is pumped through an external loop with filter units that are usually composed of hollow fibers. The tangential flow creates shear forces on the filter membrane, cleaning it and delaying filter fouling (Castilho and Medronho 2002). A derivative of TFF is alternating tangential flow filtration (ATF), where the cell suspension flow is periodically reversed. This creates a backflush effect and comes with several advantages such as reduced filter fouling, lower product retention, and inherent low shear pumping action (Karst et al. 2016; Kelly et al. 2014). Additionally, only one connection to the bioreactor needs to be made instead of two. For either approach, suitable filtration membranes must be chosen as material type and surface properties strongly influence filter fouling, product sieving and transmembrane pressure at a given permeate flux. A detailed discussion of this in the context of suspension cell processing can be found in the study conducted by Raza et al. (2024). The continued dominance of TFF and ATF-based perfusion processing of suspension cells is assisted by the available scale-down model bioreactors like the Ambr 250 from Sartorius, which make it possible to develop optimized perfusion processes using high-throughput methodologies. A closely related filtration-based perfusion solution to ATF and TFF are wave-mixed bioreactors with internal filter membranes which are available for cultivation volumes of up to 100 L. Here, the sloshing of the cell culture inside the bioreactor has a cleaning effect on the filter membrane akin to that of ATF. The simplicity of wave-mixed bioreactors with preinstalled, internal CRDs make them highly suitable for CHO seed train intensification (Malla et al. 2021) and CAR-T cell expansion processes (Dwarshuis et al., 2017).

Before the advent of TFF technology, settling-based CRDs were widespread, the simplest of which is the gravitational settler where low fluid velocities were employed to let cells sediment. As single cells are small and have only a marginally higher density than the growth medium, they have a very low sedimentation speed of 10–150 mm h^−1^ (Castilho and Medronho 2002), which is why technologies were developed to accelerate this. Notable are the application of centrifugal forces through the use of continuous centrifuges/separators and the application of acoustic resonance fields which causes the formation of cell aggregates and through this quicker sedimentation (Voisard et al. 2003). Although acoustic settlers were very popular before the 2010 s, their limited scalability and reduced performance resulted in their disuse (MacDonald et al. 2022). In the same vein, several publications reported the application of continuous centrifuges as CRDs around the turn of the century (Castilho and Medronho 2002) while continuous centrifuges are nowadays mainly used for cell washing, which, it should be mentioned, is a critical aspect of cell therapy downstream processing (DSP) (Li et al. 2021). The use of centrifugation-based CRDs for adherent cell perfusion processes is additionally hindered by the high shear forces present in the centrifuge (Gränicher et al. 2020; Hutchinson et al. 2006) which can be problematic for these shear-sensitive cells (Petry and Salzig 2021). However, alternative centrifugation approaches like fluidized bed centrifugation may fare better, if they can be turned into a CRD. While filtration-based CRDs dominate recombinant protein production processes, the larger size of cell and gene therapy products, e.g., virions, means that CRDs with inherently low product retention like acoustic settlers are making a comeback (Gränicher et al. 2020; Klimpel et al. 2025).

A major technological difference between CRDs is whether they can be installed inside or outside of the bioreactor. While external CRDs are more flexible, they have the downside of requiring some sort of pump to move the cell suspension to the CRD and back to the bioreactor, which can lead to cell damage (Yin et al. 2021) and is especially problematic for the sensitive primary cells used in the production of cell therapeutics. Additionally, it has to be ensured that the cells do not spend too much time outside the bioreactor where conditions cannot be tightly controlled and oxygen depletion may be present. This issue is especially pronounced in gravitational settlers, where cells can spend more than an hour outside of the bioreactor (Voisard et al. 2003).

As described, perfusion operation has been used actively in biopharmaceutical manufacturing for a long time. However, the rapid yield improvement of batch processing in the 90 s resulted in the industry focusing on this easier to implement manufacturing strategy (Bielser et al. 2018). In recent years, this has been changing again due to the emphasis placed on process intensification where perfusion shows high potential at many stages of USP. In fact, the cumulative process improvements achieved means that a classical 15 m^3^ fed-batch process can now be replaced with a 2 m^3^ perfusion process (Wong et al. 2022), decreasing capital expenditure and enabling the implementation of single-use technology (Zamani et al. 2018).

## Benefits of perfusion operation

In classical bioprocessing, perfusion mode operation comes with two main features. On the one hand, it can be used in suspension cell seed trains to achieve ultra-high cell densities in excess of 2 ∙ 10^8^ cells mL^−1^ (Lavado-García et al. 2022). As this corresponds to a packed cell volume of > 10% (Ott et al. 2024), not much room for improvement is left in this regard. On the other hand, the continuous outflow of clarified culture broth harvested from the bioreactor makes continuous DSP possible for secreted products. This includes therapeutic proteins like monoclonal antibodies but also more advanced products like lentiviral vectors (Singh et al. 2025) and extracellular vesicles (Gong et al. 2025).

**Fig. 2:**
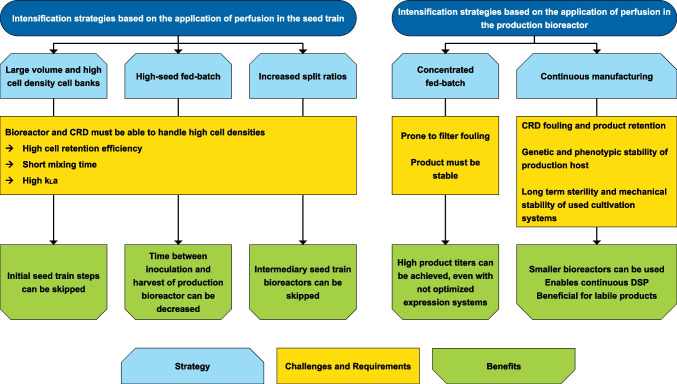
Overview of perfusion-based process intensification strategies applicable to seed train and production bioreactors

The ultra-high cell densities and therefore large cell quantities that can be produced in perfusion mode operation can be used to intensify the seed train of suspension cell processes in several ways. An overview of these strategies, including their benefits and connected challenges is depicted in Fig. 2. Through the creation of large volume, high cell density cell banks, it is possible to inoculate pilot-scale (50 L) bioreactors directly with frozen cells (Müller et al. 2022). Alternatively, the high cell densities make higher split-ratios possible. Through this, three consecutive seed train bioreactors run in batch mode can be replaced by one perfusion bioreactor at the N−1 stage whereby a 50 L N−1 bioreactor is sufficient to inoculate a 4 000 L production bioreactor (Malla et al. 2021). Another option is to use the large cell quantities produced in N−1 perfusion to inoculate high-seed fed-batch processes, where ≈ 10 × higher inoculation cell densities (2–8 ∙ 10^6^ cells mL^−1^) are used to shorten process duration while increasing product titer (Rittershaus et al. 2022), resulting in higher annual output.

The benefits of continuous processing (N-stage perfusion) are numerous. It enables the continuous harvesting of secreted, labile therapeutic products while also increasing volumetric productivities. This means that product demand can be met through bioreactors with single-digit cubic meter cultivation volumes, lowering footprint requirements, capital investments and operating costs (Müller et al. 2021). The synergy between perfusion and single-use technology is especially evident here, as smaller cultivation volumes enable the use of single-use bioreactors, which are now available in volumes of up to 6 m^3^ and offer the advantages of reduced costs and contamination risk while providing greater flexibility (Eibl et al. 2026; Samaras et al. 2022). A shift towards continuous bioprocessing is not only expected due to the mentioned benefits but also since its use has been endorsed by the U.S. Food and Drug Administration (FDA) because of the more consistent product quality attributed to this processing mode (U.S. Food and Drug Administration 2023). Since there is usually not a distinct “production phase” in therapeutic adherent cell manufacturing, the N and N−1 designations of recombinant product USP stages are not applicable. In this sense, the developed intensification approaches targeting the N−1 stage of suspension cell processing are the most promising strategies for adherent cell expansion as these align with the goal of efficient biomass production. Nevertheless, continuous manufacturing approaches should also be addressed briefly.

Continuous expansion of adherent cells is difficult to implement as a small portion of cells must be continuously detached from their growth surface while the rest needs to be kept in the exponential growth phase. One approach to circumvent the growth area constraint of regular microcarrier cultivations is bead-to-bead transfer, where cells migrate from colonized to fresh microcarriers, enabling continuous expansion without full detachment. This strategy has been shown to support extended cultures and has the potential to facilitate a quasi-continuous process mode by periodically harvesting and replenishing a portion of the microcarriers (Sion et al. 2021; Thauvin et al. 2025). Alternative approaches where cells continuously proliferate and detach from their growth substrate are discussed in the context of cultured meat production (Bellani et al. 2020). Whether this is possible to implement remains to be seen. An additional consideration with therapeutic adherent cells is their quality attributes which may change with prolonged cultivation duration and therefore needs to be regularly monitored in a continuous manufacturing scheme. Since the cultivated cells are not stable cell lines and may be subject to genetic instability and senescence, the duration of the production run is limited. While no clear consensus could be established yet, it is thought that the therapeutic quality of MSCs substantially decreases after 15–40 cumulative population doublings, with the age and health of the donor playing a critical role (Liu et al. 2020; Miclau et al. 2023). While iPSCs allow for substantially more doublings due to their self-renewing nature, a clear upper bound has not been established as increased cumulative population doublings can impact differentiation capabilities after > 30 passages (Cantor et al. 2022).

An important aspect of modern pharmaceutical manufacturing is the implementation of the process analytical technology (PAT) approach endorsed by the FDA where the aim is to assess critical process parameters and quality attributes in a timely manner to ensure a consistent, high-quality product (Wang et al. 2022). In this regard, instrumented bioreactors that feature tight pH and dissolved oxygen control in a homogeneously mixed environment are already much superior to simple multilayer cell culture flasks. More advanced in-line measurement technologies include Raman spectroscopy that can be used to monitor glucose (Graf et al. 2022) and dielectric spectroscopy for real-time viable cell density data (Sun et al. 2025). In this regard, Costa et al. found that the extracellular vesicle productivity of MSCs can be increased through low glucose conditions and showed that this can be tracked and potentially controlled using in-line Raman spectroscopy, highlighting the potential of PAT for this biopharmaceutical category (Costa et al. 2023). More specific to cell therapeutics, surface marker expression or apoptotic tendencies can be measured by on-line flow cytometry which provides insight into cell quality and can be used to determine the harvest criterion. However, the equipment costs and sampling complexity currently limit the widespread adoption of such advanced technologies in adherent cell manufacturing (Wang et al. 2022). While developing these advanced in-line analytical technologies is highly beneficial for process insight and the “quality-by-design” approach (Chemmalil et al. 2021), perfusion operation is what can be used to close control loops for nutrient and metabolite concentrations or to react to observed changes in cell quality.

## Challenges and considerations related to perfusion operation

While perfusion processing has a lot to offer, it also comes with unique challenges. The high cell densities present in suspension cell perfusion cultivations result in high oxygen demands, requiring specially designed bioreactors with a k_L_a ≥ 55 h^−1^ (Tapia et al. 2016) and short mixing times to prevent local oxygen depletion. The viscosity increase through the high cell content also makes mixing and pumping the cell suspension through external CRDs more challenging (Karst et al. 2016) and increases the shear experienced by cells (Zamani et al. 2018). That being said, due to the much lower cell densities reached with therapeutic adherent cells, oxygen supply is not the limiting factor. Instead, the challenge is growing these shear-sensitive cells in a scalable (dynamic) system in the first place. The main sources of mechanical stress are the impeller, baffles (including probes and dip-tubes), the external recirculation loop, and the CRD itself. For microcarrier- and spheroid-based cultivations, a key engineering criterion is that the characteristic turbulent length scale (Kolmogorov microscale) remains larger than the microcarrier diameter, to avoid subjecting microcarrier-cell aggregates to damaging turbulent eddies (Petry and Salzig 2021). At the same time, Petry and Salzig also showed that defined shear stress can potentially be used to keep the aggregate size below the critical diameter of ≈ 300 µm, at which nutrient diffusion limitations can start to occur. In external CRD-based systems, the pump is the dominant source of shear. Peristaltic pumps, commonly used in TFF circuits, are a well-documented source of cell lysis, whereas the ATF system’s diaphragm pump generates substantially lower shear forces. Therefore, replacing peristaltic pumps with low-shear alternatives such as levitating centrifugal pumps is an improvement (Karst et al. 2016). However, the quickly spinning impeller of such pumps can also negatively impact microcarrier cultivations by detaching the cells from their substrate (Schneider et al. 2025). Although fixed-bed bioreactors with inherent CRDs largely eliminate shear concerns because the cells are only in contact with flowing medium, harvesting is more complex. Maintaining the shear stress in a suitable window is especially important for stem cells as their quality attributes and differentiation potential can be influenced by it (Zhang et al. 2022).

As the goal of seed train intensification approaches is biomass production, it is important that the employed CRDs have high cell retention efficiencies, even at ultra-high cell densities (e.g., membrane filtration-based CRDs). Conversely, complete cell retention is not as critical in continuous processing and may even be detrimental as debris can accumulate in the bioreactor. In fact, controlled cell bleeds are usually implemented to maintain the cell density in a defined range (Pörtner 2023). A much more important aspect here is product retention by the CRD as this results in a longer residence time in the bioreactor and can diminish product quality and yield. While product retention of filtration-based CRDs can already be problematic for monoclonal antibody productions, it is even more pronounced in virus productions where retention can reach almost 100% (Göbel et al. 2024).

While perfusion mode processing can increase the volumetric productivity of the production bioreactor, it should be noted that medium preparation and storage also require considerable laboratory space. In this regard, it is important to tailor the medium composition to the cells’ needs, allowing for higher cell densities at lower perfusion rates and therefore reduced medium consumption. Additionally, by optimizing the amino acid balance, the production of harmful waste products like lactate and ammonium can be reduced (Wong et al. 2022). The naive approach to perfusion medium is to simply use batch growth medium. The next advancement is to increase the nutrient content by adding feed medium to the batch medium (Lin et al. 2017). While fed-batch processing has been the standard for recombinant protein production for many years (Xu et al. 2023), not many feed medium formulations are currently available for adherent stem cells, making this approach difficult. Nevertheless, inexpensive medium formulations for iPSC expansion are heavily researched with the current focus being on reducing the medium exchange frequency by creating more stable medium formulations through the implementation of heat-stable growth factors (Kuo et al. 2020). While these medium formulations are not specifically optimized for perfusion processing, richer and more stable formulations should make it possible to lower the perfusion rate and therefore medium consumption.

As medium costs are a principal cost driver in adherent cell therapy manufacturing, medium performance, consumption and cost per liter are critical. In this regard, Bandeiras et al. showed that xenofree medium supplementation substantially lowers the cost per dose for MSCs through improved cell yield and shorter process time (Bandeiras et al. 2018). A method used in suspension cell processing to lower the consumption of expensive growth medium is targeted nutrient supplementation where only the rapidly consumed, growth-limiting substrates, most prominently glucose and to a lesser extent glutamine, are added in concentrated form while the bulk perfusion rate is kept low (Lin et al. 2017; Yang et al. 2016). For adherent stem cells, however, dedicated single-nutrient feeding regimes comparable to the glucose-driven strategies used for suspension CHO processes are not yet established in the literature, as their nutrient consumption profiles and the stability of the relevant medium components remain poorly characterized.

A key parameter in perfusion processing is the perfusion rate as it is tightly linked with substrate supply and waste removal. While there are several ways to define it, the most common approach is to specify it as the number of **v**essel **v**olumes pumped through the bioreactor per **d**ay which is given the unit vvd (equivalent to L L^−1^ d^−1^) and is designated the dilution rate. In therapeutic adherent cell processing, the first approach to define a perfusion rate is to convert the medium exchange percentage and frequency of repeated-batch processing into a continuous perfusion rate. As medium exchanges of 50–100% are typically conducted every few days to daily, this translates to perfusion rates of 0.1–1 vvd. However, as the cell density increases with cultivation duration, static perfusion rates do not utilize the medium’s nutrients efficiently and should be adjusted based on the culture’s needs. As substrate consumption and metabolite production are directly linked to cell concentration, more advanced perfusion strategies make use of the cell-specific perfusion rate (CSPR) which specifies the volume of medium that is provided per cell and day and is commonly stated in the unit pL cell^−1^ d^−1^ (Pörtner 2023; Ozturk 1996). By keeping the CSPR constant, a consistent nutrient supply can be ensured throughout the whole cultivation duration. However, to properly implement this, real-time cell density measurement, or at least an estimation thereof, is required, which is offered by modern PAT solutions. For suspension cells, this is straightforward nowadays thanks to impedance spectroscopy. However, for adherent cells, direct in-line measurement of cell concentration is more challenging and is not always possible, depending on the bioreactor type, requiring the use of other strategies like metabolism-based (e.g., lactate production) soft-sensors (Mennan et al. 2019). Finding the minimal viable CSPR for a cell–medium combination is central to the economic viability of a production process as this directly determines the medium usage of the process. In the same vein, richer, more stable medium formulations that sustain adequate cell growth at lower perfusion rates also directly reduce medium consumption and the associated costs and logistical burden of large-volume medium preparation and storage.

An alternative way to define the perfusion rate in adherent cell cultures is the growth surface-specific perfusion rate (units: mL cm^−2^ d^−1^) which is more meaningful than the dilution rate because growth area correlates better with biomass than cultivation volume does. Especially the process transfer between dissimilar bioreactor types with different growth surface to volume ratios can be facilitated through this (Teale et al. 2025). At this point, it is worthwhile to highlight the effect different surface area to medium volume ratios have on perfusion and how this compares to the repeated-batch processing mode. Fixed-bed bioreactors, in particular, can operate with high surface area to volume ratios. Compared to microcarrier-based cultivations, identical surface- or cell-specific perfusion rates correspond to much higher dilution rates. Through this, the medium’s residence time in the bioreactor is reduced, which may increase the activity of unstable medium components while accumulated metabolites can be removed more efficiently. Regarding nutrient replenishment and waste removal, the traditional, instantaneous (partial) medium exchange remains the most efficient method, based on medium consumption. Contrary to perfusion, no fresh medium is lost through a continuous outflow. However, while efficient, instantaneous medium exchanges result in step-like changes to the cells’ environment which can have negative impacts (Kropp et al. 2016). An additional downside of efficient waste product removal is that beneficial autocrine factors are also removed alongside, which can be especially problematic when cultivating primary cells (Vis et al. 2020). In conclusion, through the implementation of PAT-based closed-loop control strategies, the robustness of perfusion processes can be substantially increased since a consistent nutrient supply can be ensured in a resource-efficient way. The automated response to process deviation is additionally particularly relevant in GMP-compliant closed systems, where manual interventions are both operationally costly and represent a contamination risk.

## Definition and classification of cell therapeutics

Cell therapeutics are a heterogeneous group that can broadly be categorized by their cell type into stem cells (e.g., hematopoietic stem cells (HSCs), MSCs), tissue specific cells (e.g., skin cells, chondrocytes), and blood cells (e.g., leukocytes, erythrocytes, and T cells) (Wang et al. 2021). As different grouping options, e.g., target or tissue of origin based, exist, this categorization is not clear cut. This is further complicated by the fact that treatments based on minimally processed cells like CD34 + selected hematopoietic stem cells are considered transplants and not cell therapies, making them exempt from the advanced therapy medicinal products (ATMP) regulation (Passweg et al. 2025). Over all categories, the vast majority of cell therapeutics, FDA-approved as well as in clinical development, are based on hematopoietic stem cells targeting bone marrow disorders and CAR-T cells for cancer treatment (Acharya et al. 2025; El-Kadiry et al. 2021).

The “stem cell” category globally contained 21 approved products in 2021 (Wang et al. 2021) and increased to 27 products by 2025 (Acharya et al. 2025). Acharya et al. reported that of the approved products, 16 are based on hematopoietic, 10 on mesenchymal and one on limbal stem cells. Of the 800 active clinical trials in 2025, the majority focused on HSCs and MSCs (Acharya et al. 2025). In the scope of this mini review, mainly the adherently growing stem cells are of interest. In this subgroup, the main players are MSCs, which can be used to treat a variety of diseases including cardiovascular diseases, graft versus host disease, degenerative disorders, and inflammatory bowel disease (Wang et al. 2021), and iPSCs, which have an even broader application range since they can be brought to differentiate into virtually any cell type (Takahashi 2025). As of 2025, 346 clinical trials utilized MSCs while only six active trials were reported for iPSCs (Acharya et al. 2025). Despite this, Japan’s health ministry recently approved the first iPSC-based cell therapeutics, one targeting Parkinson’s disease and one ischemic cardiomyopathy (Mullard 2026).

As parenteral application is the norm for cell therapeutics, it is critical that the risk of chemical (e.g., leachables) and biological (e.g., viruses and bacteria) contamination is reduced as much as possible to yield a safe product. This is complicated by the fact that the available DSP purification steps for cells are mainly limited to washing and formulation. Since end-sterilization is not possible for living cells, the whole production process needs to be conducted under qualified aseptic conditions (European Commission 2017), which is why functionally closed, single-use facilities are considered the only way forward (Nogueira et al. 2021). An additional challenge is that the source materials from which cells are isolated are not sterile. In the case of adipose-derived MSC manufacturing, it was found that 40% of lipoaspirates were contaminated with microorganisms (Szabłowska-Gadomska et al. 2023). As it is not possible to sterilize the starting material without harming the cells, the only option is to use antifungal and antibacterial additives during isolation and applying stringent microbial monitoring strategies.

In the same regard, the growth media used for the production of parenteral therapeutics should be chemically defined or at least xenofree to reduce potential adverse effects caused by medium traces left in the final product. Additionally, the medium composition can influence key quality attributes of the cultivated cells. Zhao et al. investigated serum alternatives for the cultivation of umbilical cord-derived MSC for clinical applications and found that the medium composition influenced morphology, cytokine secretion profile and differentiation potential, which in turn could impact therapeutic outcome (Zhao et al. 2024). It is therefore important that good manufacturing practice (GMP) compliant medium compositions are already used in preclinical studies. While the shift to serum-free media for MSC cultivation has already started at the turn of the century, the human platelet lysate, which was used to replace the fetal bovine serum, also suffers from batch-to-batch variability and potential contamination with pathogens. Therefore, increased efforts have been made in the last decade to create fully defined media whereby complex extracts are replaced by selected recombinant proteins like various growth factors (Huynh et al. 2026). While several xenofree/chemically defined media are now commercially available for MSC propagation, their formulations are not public knowledge, and it is difficult to verify the claims of the manufacturers regarding their strict adherence to chemically defined compounds.

## The current state of perfusion processing in therapeutic adherent cell manufacturing

While perfusion processing is a well-established intensification strategy for suspension cell cultivations, there is still a big gap for adherent cell cultivation with very limited literature describing the application of “true” perfusion. Nevertheless, it could be argued that perfusion processing is already the standard for adherent cell cultivation as the often employed “repeated-batch” process mode which is characterized by periodic partial medium exchanges represents a discontinuous quasi-perfusion. While medium exchanges are easy to implement in traditional small-scale systems like T-flasks, the required liquid handling is exponentially more challenging at larger scales and achieving medium exchange rates > 1 vvd is only possible through multiple bioreactor manipulations per day, as at most 100% medium can be exchanged at once. In the case of microcarrier- or spheroid-based cultivations, some form of cell retention is also required for repeated-batch processing to avoid extended settling times. Ultimately, perfusion mode operation is nothing else but a continuous, automated medium exchange strategy which is already the standard for bench-top dynamic iPSC cultivations, where frequent medium exchanges are required (Abecasis et al. 2017; Manstein et al. 2021; Schneider et al. 2025b). An additional advantage of perfusion mode operation is that contrary to traditional medium exchanges, the growth medium does not need to be prewarmed which greatly facilitates its implementation at larger production scales. A summary of the perfusion studies reported in this review is shown in Table 2.

**Table 2:** Key parameters of cited perfusion studies sorted by the employed growth substrate. For studies where multiple cultivation runs were conducted, the best performing ist reported. In the case of secreted products, its yield has been reported instead of the cell yield

Cell type	Reactor type	Growth substrate	CRD type	System volume [L]	Surface area [cm^2^]	Perfusion rate [vvd]	Max cell yield [cells]	Max expansion factor [-]	Cultivation duration [d]	Source
iPSC	STR	Aggregates	Internal porous 20–40-µm filter	0.15	N/A	0.8	4.28 ∙ 10^8^	5.7	7	Kropp et al. 2016
iPSC	STR	Aggregates	Internal porous 20–40-µm filter	150	N/A	1	5.25 ∙ 10^9^	70	7	Manstein et al. 2021
iPSC	STR	Aggregates	Submerged 10-µm stainless-steel filter	0.2	N/A	1.3	9.40 ∙ 10^8^	19	6	Abecasis et al. 2017
iPSC	STR	Aggregates	Submerged 10-µm stainless-steel filter	0.2	N/A	1.3	2.00 ∙ 10^8^	4.6	5	Isidro et al. 2021
Equine MSC	Hollow-fiber	Hollow-fiber	Inherent	200	17 000	N.R	2.97 ∙ 10^8^	20	7–9	Duysens et al. 2024
MSC	Mini-bioreactor (Mobius Breez)	Microcarriers	Internal filter membrane	0.002	50	0.5	3.50 ∙ 10^6^	28	7	Krupczak et al. 2024
MSC	Spinner flask	Microcarriers	Spiral microfluidic device	0.05	220	1	8.00∙ 10^6^	3.2	7	Yin et al. 2021
iPSC	STR	Microcarriers	Dip-tube covered by 70 µm mesh	1.3	4 680	0.66–2	1.70 ∙ 10^9^	21.5	4–5	Schneider et al. 2025b
iPSC	STR	Microcarriers	Proprietary microcarrier retention filter	3	7 200	1	5–15 ∙ 10^9^	44–127	9–17	Pandey et al. 2019
MSC	STR	Microcarriers	ATF (microcarrier version)	0.4	6 912	0.2	1.48 ∙ 10^8^	14.6	14	Cunha et al. 2015a
MSC	STR	Microcarriers	Dip-tube with 10-µm stainless-steel filter	0.4	2 880	0.25	2.00 ∙ 10^8^	18.5	11	Dos Santos et al. 2014
MSC	STR	Microcarriers	Internal settling tube	0.2–1.1	2 000–4 000	0.48	7.00 ∙ 10^8^	60	9–10	Sion et al. 2021
MSC	STR	Microcarriers	ATF	1.8	6 480	0.5–1.25	4.66 ∙ 10^9^	55	6	Schneider et al. 2025
MSC	STR	Microcarriers	TFF	1.8	6 480	0.5–1.5	4.54 ∙ 10^9^	40.7	7	Schneider et al. 2025
iPSC-derived MSC	Fixed bed	Non-woven PET mesh	Inherent	0.75	20 000	0.67	*1.2 ∙ 10*^13 *^	N.R	14	Gong et al. 2025
HEK293T	Fixed-bed	Non-woven PET mesh	Inherent	20	100 000	1	*3 ∙ 10*^*11 †*^	N.R	7	Singh et al. 2025
iPSC	Multiplate bioreactor	Polystyrene plates	Inherent	1.6	6 120	0.7–1.4	4.10 ∙ 10^9^	35.3	4–5	Teale et al. 2025
iPSC	Fixed bed	Woven PET mesh	Inherent	2	10 000	0.9–2.8	4.60 ∙ 10^9^	19.5	5	Teale et al. 2025

*N/A* not applicable, *N.R.* not reported, *PET* polyethylene terephthalate, ^*^particles per day (extracellular vesicle production), ^†^Transducing units (lentivirus production)

## Small scale perfusion systems

While the application of perfused mini-bioreactors for adherent cell cultivation is described in literature, these systems are not intended for large scale manufacturing but for studying cell physiology, differentiation, and tissue engineering. Here, the cells are typically cultivated on scaffolds. For example, Jun et al. co-cultivated bone marrow-derived MSCs with vascular cells in perfused three-dimensional (3D)-printed bioreactors to create an organ-on-a-chip model for bone marrow (Jun et al. 2025). Similarly, Dupard et al. used 3D printing to create human bone marrow hematopoietic niche where HSC and MSC were co-cultivated in perfusion mode to study the interactions between these cell types (Dupard et al. 2023). A scale-down perfusion model bioreactor that has been used for bone marrow-derived MSC cultivation is the Mobius Breez which features a working volume of 2 mL and a filtration-based CRD. While the authors did not focus on perfusion processing, they stressed the importance of a stable and controlled environment to ensure high cell quality, which they were able to achieve using this instrumented bioreactor at a perfusion rate of 0.5 vvd (Krupczak et al. 2024).

Another small-scale spinner flask-based perfusion bioreactor is described by Yin et al. and uses a spiral microfluidics-based device for cell retention. While this CRD was developed for cultivating CHO cells, a proof of concept with bone marrow-derived MSCs was conducted. In this case, the cultivated cells grew 27% faster with a perfusion rate of 1 vvd than in a repeated-batch cultivation subjected to 100% medium exchanges every other day (Yin et al. 2021). While the microfluidics-based CRD was not subject to filter fouling, it cannot easily be scaled-up to production size. Nevertheless, the authors claim that cultivation volumes > 100 L should be achievable through scale-out approaches.

## Perfusion in STRs

**Fig. 3:**
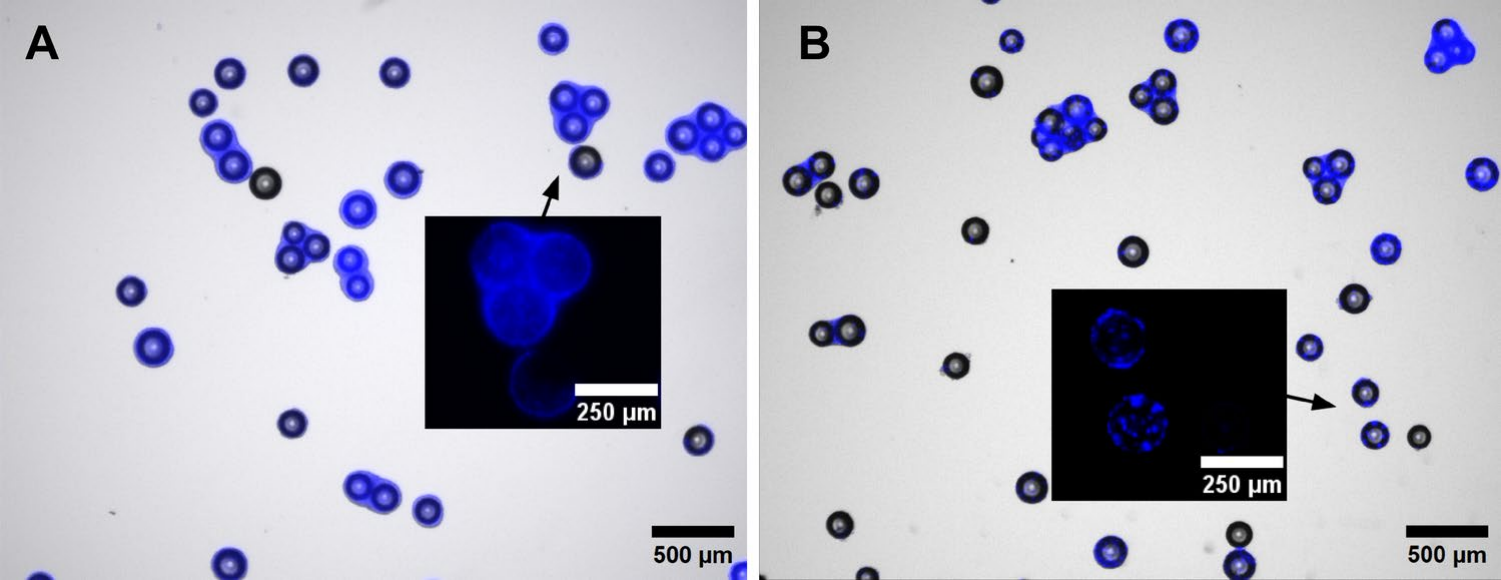
Brightfield microscopy image of adherent cells (**A** iPSC, **B** MSC) grown on microcarriers in STRs. The blue overlay corresponds to the cell nuclei stained with 4′,6-diamidino-2-phenylindole

True perfusion cultivations with the aim of adherent cell manufacturing have mainly been conducted with MSCs and iPSCs in stirred tank bioreactors (STRs). While both cell types can be cultivated on microcarriers, iPSC can also be grown as aggregates as they do not suffer from contact inhibition and are therefore not limited by the available growth surface like MSCs. Conversely, a limiting factor in aggregate cultivations is constraining the aggregate diameter to < 300 µm, as diffusion limitations can lead to necrotic cores otherwise (Wu et al. 2014). Figure 3 shows exemplary pictures of iPSCs and MSCs cultivated on microcarriers. While the iPSCs are closely packed and grow in multilayers, the MSCs are spread out more evenly, making it possible to distinguish individual cell nuclei.

In an iPSC aggregate cultivation, Manstein et al. were able to achieve cell concentrations of up to 35 ∙ 10^6^ cells mL^−1^ in a 150 mL STR fitted with an internal porous filter (20–40 µm pore size) as the CRD (Manstein et al. 2021). In a similar setup, Kropp et al. reported 47% higher cell yields for iPSCs cultivated in perfusion mode instead of repeated-batch mode. This and additional cell quality improvements were credited to the more homogeneous process conditions achievable through perfusion (Kropp et al. 2016). Similarly, Abecasis et al. highlighted the importance of the stable environment provided by perfusion which allowed them to reach 47 ∙ 10^6^ cells mL^−1^ in a 200 mL iPSC aggregate cultivation where a submerged 10-µm stainless steel filter was employed as the CRD (Abecasis et al. 2017). While Manstein et al. attempted a scale-up to 500 mL cultivation volume, issues with dissolved oxygen regulation resulted in suboptimal performance (Manstein et al. 2021), showing the necessity of conducting process development at more relevant larger production scales of multiple liters. Larger iPSC perfusion cultivations in STRs have been performed using microcarriers where Schneider et al. reported achieving 2.6 ∙ 10^6^ cells mL^−1^ in a 1.3 L cultivation using a dip-tube covered by a mesh as a CRD (Schneider et al. 2025b). Similarly, Pandey et al. reached 2 ∙ 10^6^ cells mL^−1^ in a 3 L cultivation using a “proprietary microcarrier retention filter” (Pandey et al. 2019). As already mentioned, directed differentiation of expanded iPSCs is an important step as these cells cannot be used to treat patients as is. In this regard, Isidro et al. described using the same setup as Abecasis et al. (2017) to first expand iPSCs as aggregates in perfusion mode for 5 d before a 28 d directed differentiation procedure to hepatocytes was started, which included complete medium exchanges as well as perfusion phases (Isidro et al. 2021).

In the field of perfusion-based MSC expansion, microcarriers in STR are an often-used combination. Dos Santos compared perfusion, fed-batch, and repeated-batch cultivations of bone marrow-derived MSCs at a 400-mL scale and used a submerged 10-µm stainless steel filter as a CRD. Perfusion operation resulted in approximately 3.5 times higher cell yields compared to the other process modes. However, it needs to be mentioned that the perfusion cultivation spanned 11 d instead of 7 d. Additionally, the authors mention that the fed-batch process has the lowest complexity, since there is no need to remove spent medium during the cultivation, which should make it the easiest to implement under GMP (Dos Santos et al. 2014). Sion et al. conducted a perfusion cultivation of umbilical cord-derived MSCs in a 1.5 L STR where an internal settling (dip) tube was implemented as a CRD. Starting on day 4, microcarriers and growth medium were added every other day to prevent growth area limitations before perfusion was started on day 7. The perfusion rate and diameter of the settling tube were chosen such that uncolonized microcarriers, possessing a lower sedimentation speed, were preferentially removed from the bioreactor while colonized microcarriers were retained. This way, 40% higher cell expansion factors than in fed-batch cultivations could be reached which was attributed to limiting toxic metabolite build-up and reduced collisions due to the removed empty microcarriers (Sion et al. 2021). Perfusion cultivations with external filtration-based CRDs were conducted by Schneider, Gopalakrishnan et al. and Cunha, Aguiar et al.; Schneider et al. evaluated the use of ATF and TFF CRDs for the expansion of immortalized adipose-derived MSCs at a scale of 1.8 L and compared this to the repeated-batch processing mode. The initial setup of all cultivations was identical before either perfusion (0.5–1.5 vvd) or daily 80% medium exchanges were started on day 2 or 3, respectively. Due to excessive shear forces in the TFF loop, cells were detached from the microcarriers but continued to grow as aggregates, albeit at a reduced rate. In case of the ATF cultivation, similar performance to the repeated-batch cultivation could be achieved while the formation of large microcarrier aggregates was suppressed, which could be beneficial in terms of nutrient diffusion and harvestability of the cells. Although both perfusion approaches did not result in higher cell yields or reduced processing time, the labor-intensive daily medium exchange of the repeated-batch processing mode could be replaced by automated perfusion operation, reducing labor cost and contamination risk. Additionally, demonstrating the applicability of off-the-shelf, scalable CRDs paves the way for implementing perfusion operation at larger manufacturing scales (Schneider et al. 2025). A relatively simple way to increase the performance of the perfusion cultivation mode beyond that of the repeated-batch would be employing higher microcarrier concentrations as this allows reaching higher cell densities with metabolic needs surpassing what is possible with daily medium exchanges. Cunha et al. also compared discontinuous medium exchange to perfusion mode operation using an ATF in 400-mL bone marrow-derived MSC cultivations. Here, identical medium renewal rates of 0.2 vvd were initiated on day 5, either continuous or through 50% medium exchanges every 2.5 d. To ensure sufficient growth surface, the microcarrier concentration was tripled on day 6 and intermittent stirring was employed over 3 d to facilitate bead-to-bead transfer. Over the 14-d cultivation duration, 27% higher cell yield could be achieved using perfusion mode. Additionally, the benefit of an automated system was highlighted as this minimized the required handling steps and associated risks (Cunha et al. 2015a).

## Fixed-bed bioreactors

A reversal of the STR-TFF combination is the hollow fiber bioreactor, where cells are cultivated inside a filter module (extra- or intra-capillary, depending on the design) with nutrients provided by medium circulating on the other side of the filter membrane. This bioreactor type enables perfusion mode operation without an additional CRD, is characterized by extremely low shear levels, and has been used for suspension (e.g., T cells) as well as adherent cells (e.g., MSCs) (Hulme et al. 2023; Nankervis et al. 2018). While substrates and metabolites can freely pass through the membrane, its molecular weight cut-off (MWCO) is typically chosen such that proteins like growth factors are retained. For example, a 17 kDa membrane able to retain interleukin 2 was chosen by Nankervis et al. to expand T cells in a hollow fiber bioreactor (Nankervis et al. 2018). Through this, the medium costs can be substantially reduced as inexpensive basal medium without growth factors can be used as a perfusion medium (Bräuchle et al. 2025). This benefit could potentially also be implemented in other filtration-based perfusion approaches as the necessary hardware has been developed for performing concentrated fed-batch with suspension cells. In this confusingly named operation mode, ultrafiltration-based CRDs are used instead of microfilters to allow perfusing the culture with medium while retaining secreted products in the bioreactor, making it possible to reach antibody titers in excess of 25 g L^−1^ (Yang et al. 2016). However, since the molecular sizes of the employed growth factors are substantially smaller than the large immunoglobulin G, membranes with lower MWCOs would be required, which would in turn increase the risk of filter-fouling. Specifically, some of the smallest peptides used in cell cultivation are insulin and the slightly larger insulin-like growth factor with molecular weights > 6 kDa (Bafico and Aaronson 2003). Similarly, endothelial growth factor has a low molecular weight of 6.1 kDa (Taylor et al. 1972) while most other growth factors of interest like for example fibroblast growth factor II are larger than 15 kDa (Bafico and Aaronson 2003). A well-known hollow fiber reactor is the Quantum Flex, manufactured by Terumo-BCT, which has been used to manufacture adherent cells and extracellular vesicles for a multitude of clinical studies, which Hulme et al. summarized well in their review.

Its popularity can be explained by its automated and closed nature which facilitates GMP compliance (Hulme et al. 2023). Duysens et al. showed that the hollow fiber bioreactor is also suitable for veterinary applications by expanding equine muscle-derived MSC which could be used to treat life-threatening gastrointestinal conditions in horses. In their best run, 2.97 ∙ 10^8^ cells could be produced in a 9-d cultivation (Duysens et al. 2024). Unfortunately, exact perfusion rates and medium usage were not reported but the perfusion rate was adjusted based on offline lactate measurements. A limitation of the Quantum Flex is that its growth surface area is at most 2.1 m^2^. While this is sufficient for autologous applications and clinical studies, large scale manufacturing is not possible. For comparison, 2.1 m^2^ of growth surface corresponds to a not-intensified 6 L STR microcarrier cultivation at a typical growth area to medium ratio of 3.5 cm^2^ mL^−1^. Using this approach, cultivation volumes of 30 L have already been demonstrated a decade ago (Schirmaier et al. 2014). An additional downside of the Quantum Flex is that the current bioreactor versions do not feature pH or dissolved oxygen control.

In addition to hollow fiber bioreactors, other hydraulically driven bioreactor designs exist, all of which make perfusion operation trivial due to the immobilized nature of the cells. Gong et al. implemented a multi-stage extracellular vesicle production process where iPSCs were first differentiated to MSCs and subsequently expanded in a 2 L STR using microcarriers in repeated-batch mode. After a cryo-preservation step, the MSCs were seeded into a 2 m^2^ fixed-bed bioreactor composed of layered non-woven polyethylene terephthalate meshes with a volume of 750 mL. Over the 14-d cultivation duration, 500 mL medium was replaced daily and extracellular vesicles were purified from the spent medium (Gong et al. 2025). For iPSC expansion, Teale et al. described the perfusion operation of two different hydraulically driven benchtop bioreactors. In one, the growth surface was provided by stacked polyethylene terephthalate meshes (1 m^2^) while polystyrene plates (0.6 m^2^) were installed in the other. Through the application of perfusion rates between 0.7 and 1.4 vvd, substrate limitations and metabolite inhibition could be prevented and > 4 ∙ 10^9^ cells could be harvested after 5 d of cultivation, corresponding to expansion factors of 20 and 35, respectively. While the multiplate bioreactor showed a higher expansion factor, its slow and limited process control called its scalability into question. In the mesh-based fixed-bed bioreactor, the main issues were cell attachment and distribution inside the fixed-bed, highlighting the sensitivity of this bioreactor type regarding seeding strategy and coating options (Teale et al. 2025).

Larger fixed-bed cultivations have been demonstrated with stably transfected adherent human embryonic kidney cells (HEK293T) for gene therapy purposes. Specifically, Singh et al. described lentiviral vector production in a 10 m^2^ mesh-based fixed-bed bioreactor with a total system volume of ≈ 20 L. Starting on day 4, a 7-d perfusion process was initiated with a dilution rate of ≈ 1 vvd and the permeate was collected for later purification. Through this regime, the process conditions could be tightly controlled, improving process output by a factor of 2.7 compared to the standard flatware process (Singh et al. 2025). While this case study proves that large scale perfusion cultivations of adherent cells are possible, cell harvesting was not investigated by the authors since not the cells, but the secreted lentiviral vectors were the product. In fact, the limited harvestability of fixed-bed bioreactors is what is currently hindering their implementation for adherent cell manufacturing. While efforts have been made to close this gap through enhanced harvesting procedures (Goral et al. 2024), it remains to be seen whether this approach will prove successful.

## Cell retention in DSP of cell therapeutics

The cell harvest, which represents the transition from USP to DSP, is substantially more complicated for adherent cells than for suspension cells. Usually, dissociating cells from their growth substrate is achieved through a combination of enzymatic and mechanical steps which can negatively influence cell quality (Francis et al. 2025). Furthermore, it has to be ensured that the growth substrate and potential fragments thereof are not present in the final product, which is especially challenging for microcarrier cultivations. Options to circumvent this issue include growing the cells as aggregates (Francis et al. 2025), using dissolvable microcarriers or employing biodegradable microcarriers (Handral et al. 2023). After dissociation, the harvested cells need to be washed to remove unwanted medium remnants, harvesting enzymes, cell debris, and large cell aggregates, before a final concentration step is conducted (Jossen et al. 2018). In allogeneic therapies in particular, cryopreservation is unavoidable to conserve the cells for future use (Schnitzler et al. 2016). The interested reader can find more details regarding the downstreaming challenges posed by microcarriers in MSC cultivations in the review by Mawji where harvesting enzymes, functionalized microcarriers, separation, and washing approaches are discussed (Mawji et al. 2022). In the case of iPSC-derived products, an additional purification step is required to remove undesired cell populations present after targeted differentiation which can lead to teratoma formation in patients. Doing this efficiently at large scales is currently a major bottleneck for iPSC-based cell therapeutics manufacturing (Sart et al. 2022).

As the sensitive cells need to retain their quality attributes throughout the DSP, which mainly consists of washing and concentration steps, scalable and fast processing methods are required. In this regard, many of the CRDs developed for perfusion processing could be employed, albeit at higher flow rates (Schnitzler et al. 2016). The application of TFF to wash MSCs previously expanded on microcarriers was investigated by Cunha et al. 2015b). As a first step, dead-end filtration was used to separate cells from microcarriers before TFF was used for cell washing and concentration, resulting in cell recoveries of > 80% with acceptable quality attributes. The authors highlight the importance of choosing suitable filtration membrane materials and process setpoint to find a tradeoff between damaging shear and excessive time the cells spend in the TFF loop. Here it was found that polysulfone performed better than hydrophilic polyethersulfone membranes which were prone to rapid filter fouling while nylon or polypropylene can be used in the preceding microcarrier removal dead-end filtration (Cunha et al. 2015b). In a later study, it was found that employing flat sheet cassettes instead of hollow fiber cartridges for TFF washing of MSCs resulted in 18% higher cell yields. Additionally, a method was developed to evaluate the influence of the DSP on the cells’ proteomes, with the aim of improving product and process understanding (Cunha et al. 2017). The dual-purpose use of filtration-based CRDs for perfusion operation and cell washing was described by Schneider et al. (2025). Here, ATF and TFF CRDs were first used for adipose-derived MSC expansion on microcarriers. At the end of the cultivation, the installed CRDs were used to remove the growth medium and wash the cells/microcarriers prior to the addition of harvesting solution. However, the process was impeded by rapid filter blockage since the washing step required much higher permeate fluxes than the previous perfusion operation. To circumvent this, using larger filtration areas and larger pore sizes has been proposed (Schneider et al. 2025). Other options for cell washing are single-use (counterflow) centrifugation-based approaches which have a long history in blood product processing (Li et al. 2021). Examples for commercial single-use counterflow centrifugation systems are the Rotea from Thermo Fisher Scientific (Li et al. 2023) and the kSep from Sartorius (Pigeau et al. 2018). The Rotea was specifically designed for cell therapy manufacturing and has demonstrated high recovery rates > 90% with MSCs under ideal conditions (Li et al. 2019). These systems offer an inherently low-shear environment, can operate in a closed configuration, and are compatible with a wide range of cell types and processing volumes. For microcarrier-based cultivations specifically, counterflow centrifugation may offer the additional advantage of being capable of selectively retaining or removing microcarriers based on their sedimentation characteristics, which could be exploited during DSP. However, to our knowledge, this method has not been thoroughly assessed yet. Similarly, while the use of counterflow centrifugation-based CRDs has not been described yet for adherent cell perfusion cultivations, they could prove to be a viable alternative to filtration-based CRDs.

## Conclusion

In the field of adherent cell cultivation, medium exchanges are a necessity with the currently available growth media which is why these cultivations are often performed in repeated-batch mode. While this works well at small scales, the required liquid handling quickly gets complicated at larger production scales. Through perfusion mode operation, these medium exchanges can be automated while the cells also benefit from a more stable growth environment. Although perfusion operation has already proven its merit in bench-top scale cultivations, it is striking that many researchers still report the use of makeshift internal filter-based CRDs of questionable scalability. While this can at least partially be explained by the high costs associated with off-the-shelf CRDs like the ATF from Repligen or Cobetter, the anticipated challenges faced during scale-up make the use of such purpose-built devices mandatory. Nevertheless, in the case of autologous cell therapies, the output of already existing bench-top cultivation systems is sufficient, and scalable solutions are not strictly required. Still, these products can also benefit from the automation and improved process control provided by perfusion processing.

An inherent challenge of adherent cell manufacturing is providing sufficient growth surface. As long as growth surface availability remains the limiting factor of cultivations, the benefits achievable through perfusion processing are limited. In this regard, efforts must be made to not only increase the total surface area but also the surface area to volume ratio of adherent cell cultivation systems. In microcarrier-based cultivations, the options are to add more microcarriers during the cultivation or to already start with an increased microcarrier concentration. While the first option relies on bead-to-bead transfer (Sion et al. 2020), the second makes it more difficult to achieve a homogeneous cell distribution on the microcarriers during seeding. Regardless of the cultivation system, homogeneous seeding is a challenge for adherent cell cultivation that grows with manufacturing scale. While the cells are mobile to a certain degree, inhomogeneous seeding cannot be completely compensated, which is especially problematic for discontinuous growth surfaces like microcarriers.

In summary, it is worthwhile to look at how other fields solve challenges similar to those faced by therapeutic adherent cell manufacturing. Specifically, GMP-compliant closed system perfusion processes have been developed for virus and protein productions with suspension cells. In these fields, the benefits of combining perfusion with single-use technologies have been recognized, and modern production processes heavily rely on single-use bioreactors and CRDs. Especially, the decreased contamination risk and therefore better patient safety profile make it a key enabling technology for cell therapy manufacturing. An additional technological overlap exists with the cultured meat industry, which places high hopes on (continuous) perfusion-based process intensification for the expansion of adherent primary cells. In the same vein, it should also be investigated whether cell washing and retention technologies developed for cell therapy DSP could also be used in USP for perfusion processing and vice versa.

## Data Availability

This manuscript is based on data reported in the cited sources. Picture raw data can be requested from the corresponding author.

## Abbreviations

*3D*: Three dimensional
*ATF*: Alternating tangential flow filtration
*ATMP*: Advanced therapy medicinal products
*CAR-T cell*: Chimeric antigen receptor T cells
*CHO*: Chinese hamster ovary
*CRD*: Cell retention device
*CSPR*: Cell-specific perfusion rate
*DSP*: Downstream processing
*FDA*: U.S. Food and drug administration
*GMP*: Good manufacturing practice
*HEK*: Human embryonic kidney
*HSC*: Hematopoietic stem cell
*iPSC*: Induced pluripotent stem cell
*MSC*: Mesenchymal stromal cell
*MWCO*: Molecular weight cut-off
*PAT*: Process analytical technology
*STR*: Stirred tank bioreactor
*TFF*: Tangential flow filtration
*USP*: Upstream processing
